# Spinal Metastases of the Vertebrae: Three Main Categories of Pain

**DOI:** 10.3390/life14080988

**Published:** 2024-08-08

**Authors:** Ruben Van den Brande, Charlotte Billiet, Marc Peeters, Erik Van de Kelft

**Affiliations:** 1 Faculty of Medicine and Health Sciences, University of Antwerp, 2000 Antwerpen, Belgium; 2 Department of Neurosurgery, AZ Klina, 2930 Brasschaat, Belgium; 3 Department of Radiation Oncology, Iridium Netwerk, University of Antwerp, 2000 Antwerpen, Belgium; 4 Department of Oncology, Antwerp University Hospital, 2650 Edegem, Belgium; 5 Department of Neurosurgery, Vitaz, 9100 Sint-Niklaas, Belgium

**Keywords:** spinal metastases, metastatic epidural spinal cord compression, stereotactic body radiation therapy, separation surgery

## Abstract

Oncologic back pain, infection, inflammation, and trauma are the only specific etiologies of chronic low back pain (CLBP) in contrast to most patients who have non-specific CLBP. In oncologic patients developing CLBP, it is critically important to perform further investigation to exclude spinal metastases (SM).The incidence of cancer is increasing, with 15.7–30% developing SM. In the case of symptomatic SM, we can distinguish three main categories: tumor pain; mechanical pain due to instability, with or without pathologic fractures; and metastatic epidural spinal cord compression (MESCC) or radicular compression. Treatment of SM-related pain is dependent on these categories and consists of symptomatic treatment, target therapy to the bone, radiotherapy, systemic oncologic treatment, and surgery. The care for SM is a multidisciplinary concern, with rapid evolutions in all specialties involved. It is of primordial importance to incorporate the knowledge of specialists in all participating disciplines, such as oncology, radiotherapy, and spinal surgery, to determine the adequate treatment to preserve ambulatory function and quality of life while limiting the burden of treatment if possible. Awareness of potential SM is the first and most important step in the treatment of SM-related pain. Early diagnosis and timely treatment could prevent further deterioration. In this review, we explore the pathophysiology and symptomatology of SM and the treatment options for SM-related pain: tumor pain; mechanical pain due to instability, with or without pathologic fractures; and MESCC or radicular compression.

## 1. Introduction

In 2020, approximately 1 out of 13 people suffered from chronic low back pain (CLBP), equating to 619 million people worldwide. This prevalence is rising swiftly; a 60% increase compared to 1990 is documented, and a further rise to an estimated 843 million by 2050 is expected [1]. In high-income countries, CLBP is even more prevalent compared to middle- or low-income countries (32.9 ± 10.0 vs. 25.4 ± 18.3 or 16.7 ± 15.7); this difference is speculated to be attributable to less access to health insurance/healthcare, higher levels of exercise, shorter height, and higher pain thresholds [2]. The pathophysiology causing this CLBP is most often non-malignant. However, the incidence, survival, and prevalence of oncologic disease are increasing, resulting in more patients at risk for developing back pain caused by spinal metastases (SM). SM were often associated with a poor outcome, but due to the rapidly evolving systemic, radiotherapeutic, and surgical treatment modalities, the survival of oncologic patients with or without SM is improving [3,4,5]. Oncologic back pain, infection, inflammation, and trauma are the only specific etiologies of CLBP in contrast to most patients who have non-specific CLBP. In oncologic patients developing CLBP, it is critically important to perform further investigation to exclude SM.

SM can be asymptomatic; nonetheless, in most cases, they are a cause of CLBP. Moreover, SM can lead to spinal instability, with mechanical pain with or without pathologic vertebral compression fractures (pVCF) (12.6% of SM) or metastatic spinal cord compression (MESCC) (9.6% of SM). Both pVCF and MESCC are associated with significant morbidity and are highly impactful on patients’ quality of life (QoL). Despite this high symptomatic burden, the delay between first symptoms and diagnosis is often large (mean duration 66 days from the point at which the patient reported their first relevant symptom to a health professional, interquartile range 37–205 days) [6].

The purpose of this narrative review is to highlight the importance of prompt diagnosis of SM in this overview of the epidemiology, symptomatology, pathophysiology, and treatment modalities of back pain resulting from SM. We describe SM-related symptomatology in three main categories: tumor pain, mechanical instability, and MESCC.

## 2. Epidemiology

The World Health Organization (WHO) reports the worldwide incidence of cancer has increased by 50% over the last decade to an estimated 18 million new cases in 2018: 11.7% of all cancers being breast carcinoma (2.26 million), 7.3% prostate carcinoma (1.41 million), and 11.4% lung carcinoma (2.21 million). The WHO predicts an exponential growth of cancer, with nearly 30 million new cases in 2040 and an estimated prevalence of 80 million patients with cancer [7]. One out of six oncologic patients suffers from SM, according to clinical data; in contrast, in the end-of-life setting, even 30% of these patients have SM [5]. Combining these data, we estimate that there will be between 13.3 and 27 million patients with SM in 2040. Compared to the global incidence of CLBP of 619 million patients, there are 2–4 patients with SM for every 100 patients with CLBP. Almost two-thirds of these SM occur in patients with breast, prostate, or lung cancer [8,9,10]. Regional differences exist in the frequency of these primary tumors [7].

**Figure 1:**
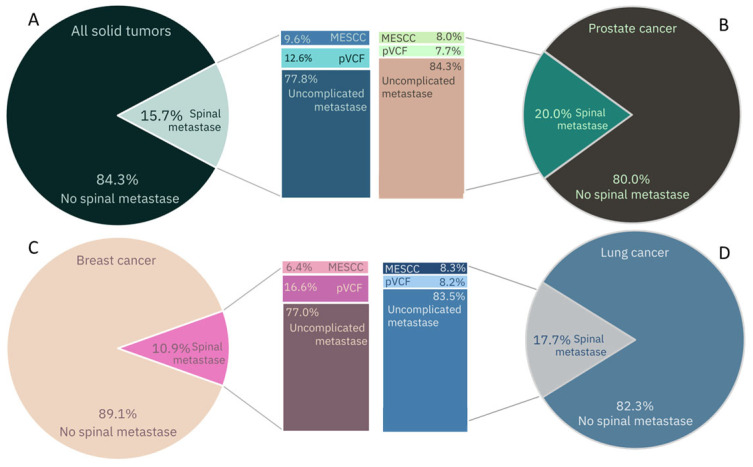
Epidemiology of spinal metastases, metastatic epidural spinal cord compression (MESCC), pathologic vertebral compression fractures (pVCF) in solid tumors. (**A**) All solid tumors. (**B**) Lung cancer. (**C**) Breast cancer. (**D**) Prostate cancer [5].

SM can be asymptomatic, cause biological pain, or result in complicated SM with either MESCC or pVCF (or a combination of these). Overall, 1 out of 8 SM causes pVCF and nearly 10% result in MESCC. In 2040, an estimated 1.33–2.7 million will have MESCC and 1.66–3.37 million will experience pVCF. Differences between primary tumor types are observed. In breast carcinoma patients, the incidence of pVCF is significantly larger compared to prostate carcinoma patients (16.6% vs. 7.7%) (Figure 1) [5]. This can be explained by the type of bone metastases; in breast cancer patients, they are typically osteolytic; in contrast, prostate carcinoma tends to lead to osteoblastic lesions [11]. In MESCC, no significant difference between primary tumor types is observed.

## 3. Symptomatology and Pathophysiology

SM as diagnosed on medical imaging can be asymptomatic in approximately 25% [12]. In the case of symptomatic SM, we can distinguish three main categories: tumor pain; mechanical pain due to instability, with or without pathologic fractures; and MESCC or radicular compression.

### 3.1. Tumor Pain

The pathophysiology of biological pain resulting from SM is a complex and multifaceted process in which the pain is not proportional to the number or size of the SM [13,14].

The bone is innervated by thin myelinated, A-δ, or unmyelinated C sensory nerves. The periosteum is the most densely innervated, by a factor 50 times denser compared to the bone marrow and 1000 times denser compared to calcified bone. Tumor cells and the inflammatory response release nerve growth factor (NGF), which promotes pathological sprouting of the sensory nerves leading to a 10- to 70-fold increase in the density of the nerve fibers and the formation of neuroma-like structures within them [12,15].

The tumor cells release a multitude of cytokines and mediators that recruit and activate inflammatory cells (T-cells, mast cells, macrophage, stromal cells); disrupt the equilibrium of osteoclasts (receptor activator of nuclear factor kappa-Β ligand (RANKL)) and osteoblasts (endothelin (ET)); and activate the nociceptive fibers of the sensory nervous system (prostaglandin E2 (PGE2), adenosine triphosphate (ATP), lactic acid and H+, ET, tumor necrosis factor (TNF) alfa, interleukin (IL) 6, -8, -15,-16, NGF, bradykinin, …), causing the pain sensation.

The activation of the inflammatory cells promotes additional release of these cytokines and mediators, resulting in secondary activation of the nociceptive fibers, leading to an increase in pain. Due to the natural circadian cortisol fluctuations, the low cortisol levels at night result in increased activity of the inflammatory cells and increased swelling surrounding the SM, causing an increase in pain with the typical nightly back pain in SM [12].

The release of RANKL, and to a lesser extent H+, activates osteoclast activity, causing a decrease in bone mineralization and impaired bone strength. The increased osteoclast activity leads to a local acidotic effect by the Warburg mechanism, protecting the osteoclasts from intracellular acidosis by releasing H+ and lactate. This leads to further stimulation of the nociceptive fibers [12,16].

The combination of these mechanisms leads to an increase in sensory nerve density, and activation of these results in the pain sensation.

### 3.2. Mechanical Pain Due to Instability with or without Pathologic Fractures

Pathologic vertebral compression fractures (pVCF) and spinal instability (without fracture) are strongly associated with pain and have a large, negative influence on the quality of life (QoL) and activity of patients [17].

The spinal instability neoplastic score (SINS) (Table 1) was developed to facilitate the estimation of fracture or instability risk. The SINS ameliorates the communication between different care providers and has great interrater reliability [18,19]. This scoring system uses six parameters to determine the risk of instability: two regarding the localization, the level of the lesion and the posterolateral involvement; one on vertebral body collapse or large involvement; one on the alignment; one on the presence of mechanical pain or occasional pain; and the last one on the type of the lesion. Lytic lesions are associated with a higher score and, thus, a higher risk of instability compared to mixed or blastic lesions [19].

SM disturb the equilibrium in the complex and interconnected process of bone formation; this can lead to an excessive proliferation and maturation of osteoclasts, leading to a loss of mineralization and the development of osteolytic lesions. These processes form a complex vicious cycle, with tumor growth and bone resorption stimulating each other [11]. Lytic lesions, zones with reduced bone mineral density (BMD), are associated with reduced bone strength and increased risk for fracture [20,21,22].

The SINS is well correlated with pain and mobility. Out of all parameters measured by SINS, mechanical pain has the highest correlation with pain and QoL [23].

### 3.3. Metastatic Epidural Spinal Cord Compression or Radicular Compression

MESCC occurs when cancer metastases in the spine grow into the epidural space and cause compression of the spinal cord. The degree of MESCC is described by the Bilsky grade 0 to 3, with 2 and 3 defined as high-grade and 1a, 1b, and 1c as low-grade. The intra- and interrater variability of this scale is excellent on T2-weighted magnetic resonance images [24].

Back pain is the most common initial symptom of SM and may be observed in as many as 88–94% of patients at the time of diagnosis. MESCC can lead to radicular pain due to the radicular compression (50% as the initial symptom and close to 80% at diagnosis) or more specific symptoms due to spinal cord compression, often with an insidious onset; ataxia (67%) is a more frequently presenting symptom compared to motor weakness, which is only the initial symptom in 40%, but close to 90% at the time of diagnosis. Sensory disturbances (30% to 75%) and/or bladder dysfunction (5 to 60% at diagnosis) may be other presenting symptoms of MESCC. Often, symptoms are slowly progressive, and patients may only seek medical assistance or get diagnosed when their mobility is affected despite experiencing symptoms for several weeks or even months [6,25,26,27]. 

The interval from the first symptoms reported to a health professional to the diagnosis of MESCC often seems too long (mean duration 66 days) [6]. Multiple studies report that approximately one-third of MESCC patients lose their ambulatory function [5]. The proportion of neurologically intact patients with MESCC decreased from 94% at three months after radiographic diagnosis to 43% at two years [28]. One may conclude that if untreated, virtually all patients with MESCC will eventually develop a neurological deficit. This loss of independence has a substantial reduction in quality of life (QoL) both in patients who are non-ambulators or with assistance, 88% and 22%, respectively. Independent ambulatory patients with SM have a QoL similar to those without SM [29].

Spinal cord injury (SCI) from MESCC is characterized by a (slow) developing chronic compression resulting in several pathophysiological processes, in contrast to an acute SCI caused by a sudden trauma. Direct cord compression and epidural venous plexus obstruction lead to compressive vasogenic edema in the spinal cord. Chronic spinal cord compression disrupts the blood–spinal cord barrier [30] (the functional equivalent of the blood–brain barrier), leading to a change in the osmotic gradient that results in increased ion and protein transport, leading to perivascular swelling and edema. The compression also induces an inflammatory response. These processes can lead to acute vascular events with ischemia and infarction, resulting in more rapidly progressive loss of function [31,32].

## 4. Treatment of Spinal Metastases-Related Pain

**Figure 2:**
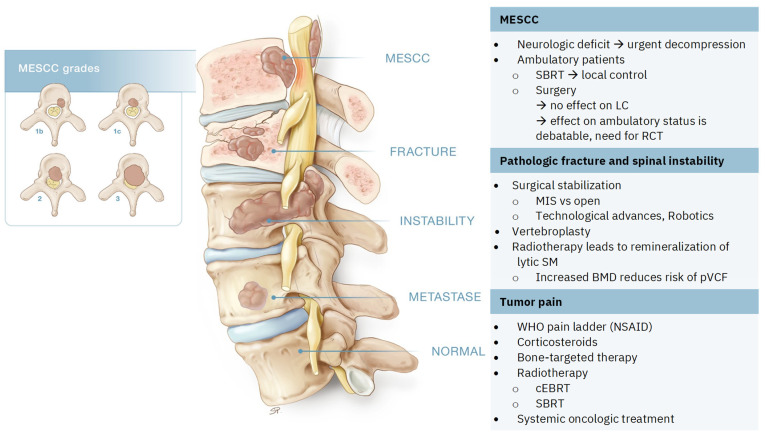
Spinal metastases (SM) and the three main categories of pain. Metastatic epidural spinal cord compression (MESCC) grades described by Bilsky et al. [24]: grades 2 and 3 are considered high grade, grade 1b and 1c are low grade. SBRT: stereotactic body radiation therapy, LC: local control, RCT: randomized controlled trial, MIS: minimal invasive surgery, BMD: bone mineral density, pVCF: pathologic vertebral compression fractures, WHO: World Health Organization, NSAID: non-steroidal anti-inflammatory drugs, cEBRT: conventional external beam radiation therapy.

As a consequence of the rapid evolution of medical advances in oncologic therapies, treatment algorithms for SM become obsolete within a few years of their creation, even before widespread implementation is reached. To overcome these shortcomings, the NOMS (neurologic, oncologic, mechanical, and systemic) framework was created. In contrast to algorithms, this framework is a dynamic decision framework that incorporates a neurologic, oncologic, mechanical, and systemic assessment of patients to guide us to optimal treatment approaches. New techniques and treatments are easily incorporated into this framework [33,34,35]. Elaborating on all aspects of this multidisciplinary framework will go beyond the scope of this review. We will focus on pain-related treatments in light of the three main categories of pain in SM (Figure 2).

### 4.1. Tumor Pain

The treatment of tumor pain in SM is multimodal, with analgesics, anti-inflammatory medication, and bone-targeted therapy to increase bone quality or reduce the pathologic increase in the density of nerve fibers. Furthermore, this symptomatic treatment, an anticancer treatment that includes local treatment such as radiotherapy or surgery as well as systemic treatment, has an important role in the treatment of tumor pain.

The WHO pain ladder should be used to reduce the pain quickly at rest as well as during movement. The use of analgesics does not reduce the risk for skeletal-related events (SREs). Non-steroidal anti-inflammatory drugs (NSAIDs) inhibit PGE2 synthesis via cyclooxygenases (COX), resulting in a reduction in PG-induced sensitization. Therefore, they are considered more useful because local inflammation is an important factor in the pathogenesis of SM-related pain. However, the evidence supporting this clinical experience is limited [31,36,37,38,39]. 

On the other hand, opioids are efficacious against neuropathic, nociceptive, and mixed pain. Various opioids with different pharmacological properties exist; there is not one superior to another in terms of efficacy [38,39]. With the use of opioids comes the risk of tachyphylaxis. This results in an increasing need for opioids to achieve the same analgesic effect. Long-term use of opioids can lead to addiction [40]. Therefore, we must be careful to preserve these medications for patients with severe pain refractory to other analgesics. It is important to be cognizant of the potential neuropsychiatric effects that opioids can have on an individual, especially for those under palliative care. By having these understandings, patient quality of life can be improved, healthcare system costs can be decreased, and patient outcomes can be met and exceeded [40].

Corticosteroids exert potent anti-swelling and anti-inflammatory effects. On one hand, the reduction in perilesional edema leads to an improvement in analgesia. On the other hand, they influence the nociceptor activation by reducing the level of pro-inflammatory cytokines and directly decreasing the pathological electrical activity of damaged peripheral nociceptive fibers; these mechanisms result in a decrease in pain intensity. The WHO strongly recommends the use of adjuvant corticosteroids in adults and adolescents with cancer-related pain to achieve pain control based on moderate-quality evidence [39]. A Cochrane database systematic review concluded that the evidence for the efficacy of corticosteroids to achieve pain control in oncologic patients is weak, despite significant pain relief that has been described in multiple studies [41]. Long-term use of corticosteroids can induce several important side effects. Therefore, the use of corticosteroids should be limited to a short period, sufficient to achieve pain control before further treatment, such as radiotherapy, can be initiated.

#### 4.1.1. Bone-Targeted Therapy

The benefit of bisphosphonates (BPs) is well proven, with significant pain prevention and reduced risk of skeletal-related events (SREs) [42,43]. BPs exert a direct apoptotic effect on osteoclasts and lead to inhibition of the development of inflammation surrounding the tumor. The WHO recommends the use of BPs to prevent and treat bone pain in adults with cancer [39].

Denosumab is a human, monoclonal synthetic antibody that inhibits the tumor-induced proliferation and maturation of osteoclasts by binding to RANKL to prevent its interaction with RANK. It delays SREs and prevents the recurrence of bone pain. Denosumab reduces the risk of SREs and improves functional outcomes more than BPs but comes with an increased risk of osteonecrosis of the jaw [44,45].

The analgesic role of Denosumab and BPs is debatable; nonetheless, these medications help to prevent pain by reducing the risk of SREs and delaying the onset of bone pain [46].

Pre-clinical studies demonstrated the ability of Tanezumab, a recombinant humanized monoclonal antibody, to bind NGF to prevent the increased synthesis of pronociceptive substances and tumor-induced sprouting and/or formation of neuroma-like structures; by these processes, it reduces the pain sensation [47]. Clinical data on the efficacy in cancer-induced pain are sparse, with only one randomized-controlled trial (RCT) comparing Tanezumab with a placebo. This study failed to show a significant difference in pain relief between these groups. In the subgroup of patients with higher baseline pain and lower total opioid use, there was a significant improvement in pain at 8 weeks [48,49]. Nonetheless, because of the limited evidence, the WHO experts have not yet been able to recommend or not recommend Tanezumab; to this date, there is no FDA (Food and Drug Administration) or EMA (European Medicines Agency) approval for Tanezumab.

#### 4.1.2. Radiotherapy

Radiotherapy is the most important local oncologic treatment modality in the treatment of tumor pain in SM. Surgery should be reserved for spinal instability, pathologic fractures, and symptomatic spinal cord or radicular compression.

Conventional external beam radiation therapy (cEBRT) is the most widespread, easily available local treatment for SM. Mostly a single dose of 8 Gy is delivered. During the last decades, the use of stereotactic body radiotherapy (SBRT) for spinal lesions has emerged. SBRT allows higher doses to the lesion while sparing the surrounding tissues and organs at risk; thus, a higher effective dose can be delivered without increased toxicity to healthy tissue. SBRT results in an increase in local control (LC) [50,51]. The results of RCTs on the pain response of cEBRT and SBRT are conflicting. Bindels et al. recently published a systematic review and meta-analysis comparing SBRT and cEBRT for painful SM. cEBRT leads to a pain response (decline of at least 2 points on an 11-point scale without an increase in opioid use) in 52% (41–64%) compared to 62% (55–68%), RR 1.22 (95% CI 0.96–1.54). There was a significant benefit for SBRT over cEBRT in complete pain response (pain score = 0) (RR 2.47 (95% CI 1.24–4.91). Further research is needed to study the associations of specific dose regimens and could be used to help identify what subgroups benefit from SBRT [52].

### 4.2. Mechanical Instability with or without Pathologic Fractures

The benefit of surgery in patients with proven instability (SINS > 13 and SINS 7–12 with mechanical pain) is well established. Surgical stabilization of lesions with SINS ≤ 6 is not indicated since there is no benefit regarding pain, QoL, or activity [17].

During the last decades, there has been a tendency towards minimal invasive surgery (MIS). Technological advances, such as computed tomography (CT)-navigated screw placement and more recently robot-assisted screw placement [53,54], are extremely helpful in the evolution of safe and MIS techniques. The use of robotic systems leads to safer placement of thoracic pedicle screws [55]. If a posterior fusion is performed, this most often includes two levels above and two below. In non-junctional levels, short constructions (cement augmented one level above and one below) show a similarly low rate of material failure compared to the more conventional two above and two below [56]. These short constructs can lead to less invasive surgery, reducing the risk of wound complications. After MIS, wound complication rates are reduced significantly compared to open procedures (6.6% vs. 11.5%; *p* < 0.05) [57].

Percutaneous vertebroplasty is an even less invasive technique. In selected cases, vertebroplasty is associated with rapid pain relief, starting immediately after the procedure, which is sustained in follow-up [58]. 

Multiple studies compared open and MIS techniques. Regarding pain relief, there is no significant difference between open and MIS. MIS is associated with reduced blood loss, lower rate of wound complications, and shorter length of stay [59]. In oncologic patients, these benefits are important, since this facilitates a swift start of systemic oncologic treatment and/or post-operative radiotherapy, leading to improved local control [60].

Despite all of these technical improvements. The result of surgery is strongly dependent on the indication. In the case of mechanical pain or proven instability, surgical stabilization leads to improvement in pain, QoL, and activity. In the absence of mechanical pain or instability, surgery has no benefit regarding these outcomes. 

The risk of surgery is increased in oncologic patients due to a nutritionally depleted status, comorbidities, and/or potential side effects of systemic therapy. Recently, two systematic reviews examined the surgical complications in degenerative spine surgery [61] and surgery for SM [62]. In surgery for SM, the prevalence of surgical site infections (SSI) is doubled compared to degenerative spine surgery (6.5% (135/2088) vs. 3.1% (603/22475)). These increased risks and consequences must be taken into account when deciding if the burden of surgery would be beneficial for the individual patient.

In the absence of proven instability or mechanical pain, there is no indication for surgical treatment. In the case of lytic lesions, radiotherapy can improve bone mineral density (BMD), resulting in improved bone strength. In contrast to the benefit of SBRT over cEBRT in local control and maybe also pain response, there is no benefit of SBRT over cEBRT in this process of remineralization. The remineralization is the largest in lytic SM in breast cancer. The only exceptions are lytic SM of renal cell carcinoma; there is no remineralization of renal lytic SM after radiation therapy. These findings should be taken into account when defining a surgical strategy in the potentially unstable group, as defined by the SINS [63].

### 4.3. Metastatic Epidural Spinal Cord Compression or Radicular Compression

If MESCC leads to a neurologic deficit, the benefit of surgery is well proven. Multiple studies have demonstrated that early intervention improves the odds of functional recovery and that being ambulatory before treatment is the best prognostic factor for retaining ambulatory function [64]. The landmark paper by Patchell et al. concluded that surgery followed by cEBRT was superior compared to cEBRT in maintaining ambulatory function [65]. As the sole RCT on this topic, the impact of this study on the number of surgeries for SM was significant [66].

Especially with the promising results of SBRT, the indication for surgery is debatable in the absence of a neurologic deficit or spinal instability. The effect of surgery can be measured in terms of local control, ambulatory function, quality of life, and complications. A recent meta-analysis demonstrated excellent local control rates of 86% (95% CI 84–88%) for SBRT compared to 60% (95% CI 60–69%) for cEBRT (*p* < 0.05) in MESCC from solid primary tumors. The effect of surgery on a 1-year LC (0.89 (95% CI 0.66–1.20)) was non-significantly different; therefore, we can conclude that not surgery but radiotherapy, in particular SBRT, provides durable LC [51]. The benefit of surgery to retain ambulatory function was not confirmed by other studies; overall, the effect of surgery on ambulatory function was non-significant (OR 1.51 (95% CI 0.83–274)). Regarding QoL, there seems to be a benefit for surgery; nonetheless, SBRT alone provides significant improvement in pain/discomfort, mobility, and usual activity; this improvement in QoL is preserved for up to 5 years [67]. The risk of complications in surgical treatment exceeds this risk in SBRT (and cEBRT) [51].

Recently, Patel et al. demonstrated excellent local control and functional outcomes in 143 patients with MESCC treated by SBRT alone, suggesting that SBRT is a reasonable approach in inoperable patients or cases unable to be successfully surgically downgraded [68]. Surgery has an important role but does not improve local control or survival in the absence of instability or neurologic deficit. In the case of high-grade MESCC in the absence of a neurologic deficit, the role of surgery is debatable, as some studies demonstrate good LC for SBRT without preceding surgery. A randomized study comparing surgery followed by SBRT and SBRT alone is needed to determine if the burden of surgery provides additional benefits for patients with MESCC [51].

## 5. Discussion

Quality of life (QoL) has emerged as a primordial outcome parameter in the evaluation of treatment effects. Quality of life (QOL) and length of life (LOL) are intertwined outcome parameters, as QOL has been demonstrated as a prognostic factor for survival [69,70]. Oncologic patients and their caregivers face difficult decisions regarding different treatment options and their respective impact on QOL and LOL. In elderly patients, QOL is often preferred above LOL, whereas in younger patients, aggressive treatment protocols tend to be chosen to increase LOL [71]. Since curative treatment is not possible in metastatic disease with SM, QoL is the driving force in deciding on treatment plans. The burden of treatment should be weighted to the benefit.

The care for SM is a multidisciplinary concern. It is of primordial importance to incorporate the knowledge of specialists in all participating disciplines, such as oncology, radiotherapy, and spinal surgery, to determine the adequate treatment to preserve ambulatory function and QoL while limiting the burden of treatment if possible. Timely diagnosis and subsequent adequate treatment can prevent further deterioration, improve pain, and retain ambulatory function in these patients.

Awareness of potential SM and defining the type of pain (biological, mechanical, and/or radicular (MESCC)) for each involved vertebra is the first and most important step in the treatment of SM-related pain. Early diagnosis and timely treatment could prevent further deterioration, for example, Denosumab or bisphosphonates preventing the progression of lytic SM and pathologic fractures; timely radiotherapy providing local control, preventing symptomatic spinal cord compression and leading to remineralization of lytic SM; or timely surgical stabilization preventing significant pathologic fractures with invalidating pain or neurologic deficit or urgent surgery to decompress the spinal cord in cases of neurologic deficit or gait disturbances.

Awareness of SM in patients with back pain prevents a delay in diagnosis and subsequent treatment. A swift start in adequate treatment prevents further deterioration and ameliorates the QoL.

## 6. Conclusions

SM-related pain can be described in three main categories: tumor pain, mechanical pain, and MESCC. Awareness of SM in oncologic patients with back pain is of primordial importance. This prevents a delay in diagnosis and subsequent treatment. A swift diagnosis and start of adequate treatment can prevent further deterioration and ameliorate the QoL. Systemic therapy and bone-modifying agents are important in the prevention of symptomatic SM or SRE. Radiotherapy leads to local control, pain control, and remineralization of lytic lesions. Surgery has an important place in treating instability and in urgent decompression in cases of neurologic deficit in MESCC. The care for patients with SM is a multidisciplinary concern due to the rapid evolutions in all disciplines, which need to be incorporated in defining the best treatment strategy.

## Figures and Tables

**Table 1:** Spinal instability neoplastic score [19].

Spinal Instability Neoplastic Score	
Component	Score
**Location**	
Junctional (O—C2; C7—T2; T11—L1; L5—S1)	3
Mobile spine (C3—C6; L2—L4)	2
Semirigid (T3—T10)	1
Rigid (S2—S5)	0
**Pain**	
Mechanical pain	3
Pain without mechanical pain	2
Pain free lesion	0
**Type of bone lesion**	
Lytic	2
Mixed (lytic/blastic)	1
Blastic	0
**Spinal alignment**	
Subluxation/translation	4
De novo deformity (kyphosis/scoliosis)	2
Normal alignment	0
**Vertebral body collapse**	
>50% collapse	3
<50% collapse	2
No collapse with >50% of body involved	1
None of the above	0
**Posterolateral involvement**	
Bilateral	3
Unilateral	1
None	0
**Criteria of instability—total score**	
Stable spine	1—6
Potentially unstable spine (indication for consulting spinal surgeon)	7—12
Unstable spine (consider surgical intervention)	13—18

## Data Availability

No new data were created or analyzed in this study. Data sharing is not applicable to this article.
